# Design of Optimization Algorithm for Configuration of Amateur Sports Training Equipment in Smart City Community

**DOI:** 10.1155/2022/9572395

**Published:** 2022-06-23

**Authors:** Yuehong Wan, Hong Tang

**Affiliations:** ^1^Physical Education College of Jiangxi Normal University, Nanchang 330022, China; ^2^School of Information Engineering, East China Jiaotong University, Nanchang 330013, China

## Abstract

Community amateur sports training equipment is necessary to ensure the development of national fitness activities. It is found that the design concept of community public sports space in China is not perfect, the structural layout is not reasonable, the stock space is not optimized, and the resource allocation is not balanced. In particular, there is a lack of indoor fitness facilities and sports venues in the community, and the people's “fitness where to go” is still a difficult problem. In the smart city construction, the resource integration and optimization of community amateur sports training facilities have also been further developed. Considering various influencing factors, this paper establishes a location model of community amateur sports training facilities with the least total cost and convenience. Aiming at the practical optimization problem with high complexity, an improved adaptive weight multiobjective particle swarm optimization (PSO) model is proposed. These parameters in the algorithm were adjusted dynamically, which balanced the overall search ability and partial improvement ability of the PSO algorithm and completed the optimal scheduling of community amateur sports training equipment configuration while ensuring the optimal global solution. Experimental results show that the algorithm's efficiency and searching ability have been further promoted. It also has an excellent performance in solving the complex location problem of community amateur sports training facilities.

## 1. Introduction

In January 2019, the CPC Central Committee clearly proposed to take people's yearning for a better life as the starting point and focus of spatial planning [1]. Improve the quality of the living environment [2]. Improve the quality of people's life [3]. Community amateur sports facilities are an important carrier for public fitness activities [4]. It is a necessary condition to ensure the development of national fitness activities. For improving national physical quality. Achieve high quality of life. The cause of national fitness continued to develop. The supply and service level of community amateur sports facilities continued to improve. The “six sides” project of national fitness has been preliminarily constructed as the main content. A relatively sound public service system for national fitness covering urban and rural areas [5]. According to the report on the development of Chinese mass sports (2014) issued by the State Administration of sports in 2014. The number of sports parks in China is 1662, according to the statistical survey data of national sports venues in 2019. In 2019, there will be 823500 national fitness paths in China. 76800 fitness trails, according to the statistical bulletin of national economic and social development in 2020 [6]. By the end of 2020, there were 3.713 million sports venues in China.

However, the effective supply of community amateur sports facilities in China is insufficient. The utilization rate of existing facilities is low. Popular and convenient amateur sports facilities are still seriously insufficient [7]. These have formed a serious contradiction between supply and demand with the growing fitness demand of the public [8]. “Land grabbing,” “noise disturbing residents,” “elderly occupying the stadium,” and other events occur frequently [9]. People's “fitness where to go” has become the focus of social attention. Hindering the development process of the national fitness project [10]. Under the tight situation of land resources, reasonable optimization of the existing community amateur sports facilities is occurred [11]. “Make the best use of everything” so that community amateur sports facilities can meet and guide people's sports and fitness needs.

Focus on the convenience and benefit of the people. China has implemented policies and measures such as the “six sided project” of mass sports. Increase the effective supply of community amateur sports facilities [12]. Community amateur sports facilities around the masses are also stepping up the layout and construction. However, the layout method of “sticking to the seams” is usually adopted according to the actual situation in the specific implementation process. There is still a lack of echo with the surrounding environment and necessary humanistic care [13]. “Basketball and fitness dance conflict case” and “fitness dance disturbing residents” were once pushed to the commanding height of public opinion. The lack of sports fitness venues and facilities and the disturbing fitness activities have fully exposed the defects and deficiencies in community amateur sports facilities [14]. The official website of the Sichuan Provincial People's government once listed “unscientific setting of square dance venues, nonstandard management and noise disturbing residents” as one of the “ten things that the masses are most dissatisfied with.” To some extent, this also reflects the unsatisfactory layout planning of community amateur sports facilities in China. The accessibility of community amateur sports facilities was not fully considered in the planning and site selection [15]. It is not enough to meet the time and space needs of different people and the needs of fitness activities with multilevel and diversified configuration [16]. Especially in the context of rapid aging, community amateur sports facilities show some problems, such as insufficient scale, unreasonable function allocation, and poor accessibility. It has seriously affected the demand for outdoor activities of the elderly [17]. It is of great significance to pay attention to improving the accessibility and convenience of community amateur sports facilities in geographical space and the safety, fitness, and entertainment of content space [18].

Health is the premise of a happy life. Health is the foundation of a country to create a better future. Health is the strength of national prosperity. National fitness is the foundation of building a sports power [19]. Achieve national health through national fitness, and then achieve the goal of a well-off society in an all-around way. It is also the internal requirement of accelerating the construction of a sports power [20].

The supply of community amateur sports facilities is insufficient and unbalanced. Insufficient supply is mainly reflected in the single supply type of venues around the masses [21]. In particular, there is a lack of indoor fitness facilities and sports health service platforms in the community. People's “fitness where to go” is still a difficult problem.

With the rapid development of China's economy and people's increasing concern for health, community amateur sports facilities, as an infrastructure for national service, are developing rapidly across the country [22]. It has had a major impact on people's lives and health. Community amateur sports facilities are the core configuration of public sports space. To a large extent, it determines the development of national fitness. Community amateur sports facilities' spatial distribution and location must be reasonable and scientific [23]. Its location optimization has always been an important planning problem in most cities. It is also an issue of concern and concern to scholars. Because of its nonlinearity, high complexity, and many constraints, the traditional mathematical model can not obtain the global optimal solution [24]. The particle swarm optimization algorithm has a strong global optimization ability [25]. The group parallel search method is used to calculate and solve, with high efficiency and fast convergence speed [26]. In this paper, the improved adaptive weight multiobjective PSO is used to solving model, which is applied to the location problem of community amateur sports facilities with constraints, and good results are obtained.

## 2. Methodology

### 2.1. User Satisfaction Model

In order to improve users' satisfaction and fitness experience during the construction of community amateur sports facilities, users' satisfaction is judged by the distance of community amateur sports facilities. According to the questionnaire survey, the satisfaction is shown in Table 1.

Therefore, if you want to improve user satisfaction, you need to ask for the time period of going to the fitness place [*S*_*g*_, *F*_*g*_]. The longer the journey time, it will directly reduce user satisfaction. There is a negative correlation between user satisfaction and journey time; that is, the longer the journey time, the worse the user satisfaction will be. The satisfaction function is shown in the following formula:

(1) $$\begin{array}{c} S_{g}=1-\frac{S_{g}^{d}-S_{g}^{r}}{F_{g}-S_{g}}, \end{array}$$

where *S*_*g*_^*d*^ is the ideal travel time and *S*_*g*_^*r*^ is the actual travel time. At the same time, the AHP is used to determine the key degree of amateur sports facilities in each community to users. The weight of facility *g* is recorded as *M*_*g*_, and the total user satisfaction of all facilities is recorded as shown in the following formula:

(2) $$\begin{array}{c} S=\sum_{g\in G}S_{g}\cdot M_{g}. \end{array}$$

In order to let users experience the practicability and economy of the community amateur sports facilities optimization model, the total satisfaction acceptable to users is recorded as *A*, combined with the lowest cost model.

(3) $$\begin{array}{c} \sum_{g\in G}S\cdot M_{g}>A. \end{array}$$

### 2.2. User Demand Fluctuation Model

Compared with users, the main purpose of optimal configuration is to realize the use demand of fitness equipment. In this topic, the use frequency of equipment is selected as the fluctuation degree of user demand. Therefore, if the fluctuation degree of user demand is smaller, the use frequency of fitness equipment is more stable. Divide the day into 48 time periods, each of which is 30 minutes. The optimization objective function is shown in the following formula:

(4) $$\begin{array}{c} d=\underset{1⩽n⩽N}{\max}\overset{G}{\underset{g=1}{\sum }}e_{g}^{h}-\underset{1⩽n⩽N}{\min}\overset{G}{\underset{g=1}{\sum }}e_{g}^{h}, \end{array}$$

where *e*_*g*_^*h*^ is the usage frequency of facility *g* in period *h*. *G* is the total number of facilities. *g*=1 indicates the first facility. The smaller the final result, the smaller the fluctuation of user demand.

### 2.3. Multiobjective Processing

The multiobjective optimization is processed by the method of square sum weighting, and the optimal value *f*_*x*_ of each objective function is obtained, respectively. Since it is impossible to achieve the optimal situation of each objective at the same time and try to ensure that it approaches the ideal situation, the weighted evaluation function is constructed as follows:

(5) $$\begin{array}{c} F\left(I\right)=\sum_{x=3}^{t}M_{x}{\left[\frac{f_{x}\left(I\right)-f_{x}}{f_{x}}\right]}^{2}. \end{array}$$

The optimal solution of multiobjective optimization is obtained through (5). *M* is the weight coefficient, which can reflect the importance of each objective in the optimization process. Based on this, the unified function of the multiobjective optimization problem in this paper can be constructed.

(6) $$\begin{array}{c} F\left(I\right)=M_{C}\left(\frac{C_{\min}}{C_{\min}\Delta }-1\right)+M_{S}\left(\frac{S_{\max}}{S_{\max}\Delta }-1\right)+M_{d}\left(\frac{d_{\min}}{d_{\min}\Delta }-1\right), \end{array}$$

where *C*_min_Δ, *S*_max_Δ, and *d*_min_Δ represent the optimal value of facility cost, user satisfaction, and fluctuation degree of user demand, respectively.

### 2.4. Standard PSO Algorithm

PSO is a classical intelligent algorithm based on mutual cooperation to find the most appropriate solution [27]. Compared with other algorithms, it does not have too many complex parameters and is easier to implement. In this text, a multiobjective PSO algorithm (IAW-MOPSO) with an improved weight strategy is proposed to consider the fitness of the whole particle. It can not only solve multiple objective optimization problems but also optimize faster. At the same time, in the search principle of the optimal solution, it combines the idea of nonsupported sorting and the method of Pareto optimal appropriate allocation and uses the cooperative relationship between particles to constantly update the optimal solution of particles, so as to solve the problem. Finally, the particle set with the best fitness is selected.

The core of the PSO algorithm is to determine the overall best value and local best value of the particle by changing the next movement through its own experience and the better in the population [28]. The update process is shown in the following formula:

(7) $$\begin{array}{c} \left\{\begin{array}{c} q_{x+1}=mq_{x+1}+c_{1}\cdot \text{rand}\cdot \left(U_{{\text{best}}_{x}}-I_{x}\right)+c_{2}\cdot \text{rand}\cdot \left(a_{{\text{best}}_{x}}-I_{x}\right),\\ I_{x+1}=I_{x}+q_{x+1}, \end{array}\right. \end{array}$$

where *q*_*x*_ is the velocity of particles. *I*_*x*_ is the position of particles. *U*_best_*x*__ is the best position found so far for each particle. *a*_best_*x*__ is the best position in all groups. Rand() is a random function between 0 and 1. *q*_*x*+1_ is the *x + *1 particle velocity. *I*_*x*+1_ is the position of the *x + *1 particle. *c*_1_ and *c*_2_ are learning factors. *m* is the dynamic weight value of particle swarm. Update inertia weights with nonlinear variations as follows:

(8) $$\begin{array}{c} m_{v}=\frac{m_{\max}-\left[v\left(m_{\max}-m_{\min}\right)\right]}{n_{\max}}, \end{array}$$

where *v* is the current iteration number. *m*_*v*_ is the weight value updated in the *v* iteration. *m*_max_ and *m*_min_ are the maximum and minimum values of *m* set artificially. *n*_max_ is the maximum iteration number.

### 2.5. Improvement of PSO Algorithm

Compared with other intelligent algorithms, the PSO algorithm is simpler to set parameters in the process of solving problems, and simpler to understand compared with other bionic algorithms. The difference is that the PSO algorithm does not need coding, so it does not have the solving accuracy limitation brought by coding in other bionic algorithms. However, it still has the problem that the universal intelligent algorithm is better for single objective optimization and easy to fall into some of the most optimal when carrying out multiobjective optimization. To solve this problem, this paper promotes the standard PSO. It not only dynamically adjusts the inertia weight and learning factor but also introduces dynamic time-varying control factors to restrict the position update amplitude of particles, because the nonlinear time-varying adjustment of parameters can obtain better algorithm performance than linear adjustment. These parameters are adjusted by nonlinear dynamic adaptive time-varying adjustment strategy. The inertial weight *m* is the most important parameter in the multiobjective PSO. With the increase of *m*, the overall search ability of the algorithm will be promoted, and with the decrease of *m*, the part search ability of the algorithm will be promoted.

The improved PSO algorithm optimizes the configuration of community amateur sports training equipment, and the management unit can adjust the community amateur sports training facilities on this basis. The improvement process of this algorithm is as follows:

(1) Calculate the current fitness value of all particles
(2) Update and iterate the new generation of particles and count the particles whose fitness value is more than and less than the average value in the population
(3) Using nonlinear dynamic inertial weights to update *m*, the global and local searching ability of PSO is improved
  (9) $$\begin{array}{c} m=\left\{\begin{array}{cc} m_{\min}-\frac{\left(f_{\text{mean}}-f_{\min}\right)\Delta m}{\left(f_{q}-f_{\min}\right)}, & f_{\text{mean}}\leq f_{q},\\ m_{\max}, & f_{\text{mean}}>f_{q}, \end{array}\right. \end{array}$$
where *m*_max_ and *m*_min_ show the maximum and minimum values of *m*, respectively, Δ*m*=(*m*_max_ − *m*_min_). *F*_mean_ show the current particle fitness value. *F*_*q*_ show the current particles average fitness value. *f*_min_ show the current particles minimum fitness value. *m* changes with the fitness value of particles.

When the *f*_mean_ of the particles tends to be uniform, or vice versa. The particle whose *f*_mean_ is better than *f*_*q*_ and its corresponding *m* is smaller, which protects the particle. For the particle below *f*_*q*_, its corresponding *m* is larger, so that the particle will move towards a better search area.

### 2.6. Dynamic Parameter Update Strategy

In particle swarm optimization, inertia weight *ω* function of is to keep the particle in the motion state of the previous moment, and its value has an important influence on the convergence of the algorithm. The basic PSO algorithm sets the inertia weight as a fixed value, and its search result is very poor. The main function of the learning factor is to adjust the proportion of individual optimal position and overall optimal position in speed update to balance the overall search ability and part search ability of the algorithm. In order to make the algorithm more flexible and reliable, this paper adopts the parameter dynamic adjustment strategy. The following formula conveys all the details:

(10) $$\begin{array}{c} \omega =\omega_{\max}-\frac{\left(\omega_{\max}-\omega_{\min}\right)n}{N_{\max}},\\ c_{1}=c_{\max}-\frac{\left(c_{\max}-c_{\min}\right)n}{N_{\max}},\\ c_{2}=c_{\min}+\frac{\left(c_{\max}-c_{\min}\right)n}{N_{\max}}, \end{array}$$

where *ω*_max_ show the maximum value of inertia weight, respectively.  *ω*_min_ show the minimum value of inertia weight, respectively. *N*_max_ show the maximum number of iterations. *C*_max_ show the maximum value of learning factor, respectively. *C*_max_ show the minimum value of learning factor, respectively. At the initial stage of the algorithm iteration, the inertia weight value is large, the initial speed of particles changes rapidly, the algorithm has a fairly strong overall search ability, the learning factor *c*_1_ value is large, and the *c*_2_ value is small, so the algorithm has a fairly strong individual cognitive ability. With the iteration of the algorithm, the inertia weight decreases, the change of particle velocity slows down, the value of learning factor *c*_1_ decreases and the value of *c*_2_ increases. The algorithm has a strong global cognitive ability.

**Figure 1:**
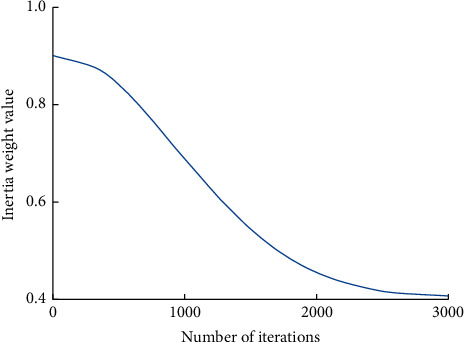
Inertia weight value change curve.

**Figure 2:**
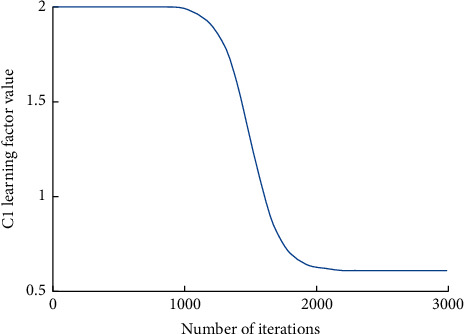
*C*_1_ learning factor value change curve.

**Figure 3:**
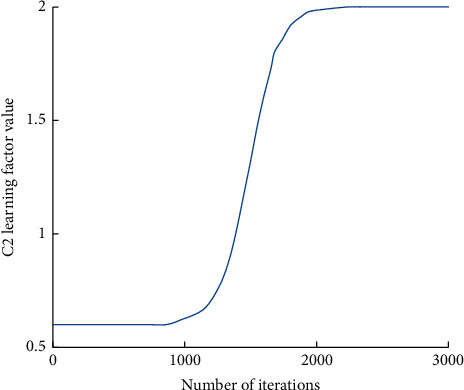
*C*_2_ learning factor value change curve.

The variation curve of each parameter is shown in Figures 1–3.

### 2.7. Model Solving Process

Aiming at the optimal allocation strategy of amateur sports training equipment in the smart city community, this paper studies the improved adaptive weight multi-objective PSO algorithm.

**Figure 4:**
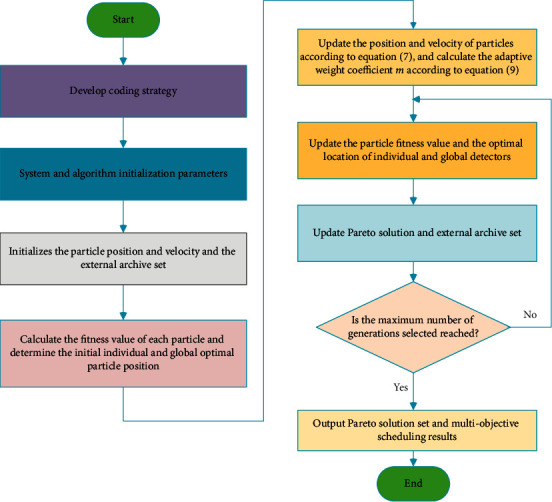
Improved MOPSO algorithm flow chart.

When solving the multiobjective problem, the optimal individual can be selected through the Pareto hierarchical sorting principle, and finally, the Pareto optimal solution set can be obtained. The specific multiobjective scheduling operation process is as follows:

  Step 1: coding strategy. The coding strategy adopts binary coding, and each particle represents a feasible solution of the multiobjective problem. All particles are designed as “gh” dimension 0–1 matrix *I*=(*i*_*xy*_)_*gh*_, where *i*_*xy*_ represents the *x* flexible load, and the *y* period is selected as the working period, ∑_*y*=1_^*g*^*i*_*xy*_=1,  *x*=1,2,…, *h* which means that each equipment selects a period for operation. The particle velocity is expressed by *q*=(*q*_*xy*_)_*gh*_.
  Step 2: system and algorithm initialization. Determine the load model to be dispatched, and inputting the operation time, power, model, and other basic parameters of each load. Setting the particle swarm size as *U*_size_, the maximum number of iterations, *n*_max_ and other algorithm parameters, initialize the particle swarm and the external file set at the same time, and each particle is randomly distributed in the feasible solution space and given an initial velocity within a given range.
  Step 3: determining extremal value. The fitness value of each particle was calculated. The historical optimal position was recorded as the individual extreme value. The global optimal solution was found in the external archive set. The best solution was selected as the global extreme value by comparison with the individual optimal solution.
  Step 4: update the velocity and displacement of particles according to equation (7).
 
*Step 5*: update the weight according to equation (9).
  Step 6: update location. According to the fitness updating historical best *U*_best_ and overall best *a*_best_, the current particle fitness value is compared with the previously determined optimal solution. If the current optimal solution is better, the extreme value is replaced.
  Step 7: update the external file set. Retaining the currently found optimal solution set, then merge the solution set found in the latest iteration with the current optimal, and selecting the nondominated optimal again.
  Step 8: judge whether the termination conditions are met. If the conditions are met, the algorithm terminates the iteration and outputs the optimal solution set, the optimized community amateur sports training equipment and facilities. Conversely, return to step 4 to continue optimization as shown in Figure 4.

## 3. Result Analysis and Discussion

To prove the algorithm's effectiveness in solving complex and multiconstraint problems in reality, it is applied to solve the location of community amateur sports training facilities. The location coordinates of 8 communities are collected. The coordinates of each community and the total amount of fitness needs are shown in Table 2.

It is planned to build amateur sports training facilities within the range of 200 ≤ *x* and *y* ≤ 3000 to minimize the initial construction cost and the total cost of users. To meet the timeliness requirements of users' fitness, it is agreed that the maximum distance between sports training facilities and the community shall not be greater than 2000 m. The use loss cost is set as 2 yuan/person time. The construction cost is different in different subregions: within the coordinate range of 200 ≤ *x, y* ≤ 1500, the construction cost is 30000 yuan. Within 1500 ＜ *x, y* ≤ 3000 coordinates, the construction cost is 35000 yuan; within the coordinate range of 200 ≤ *x* ≤ 1500, 1500 ＜ *y* ≤ 3000, the construction cost is 40000 yuan. Within the coordinate range of 1500 ＜ *x* ≤ 3000, 200 ≤ *y* ≤ 1500, the construction cost is 25000 yuan. According to the given constraints, the optimal location coordinates of community amateur sports training facilities are determined by using the proposed improved PSO optimization model to minimize the total operation cost. The distribution of construction costs is shown in Table 3. The total operating cost analysis is shown in Table 4.

**Figure 5:**
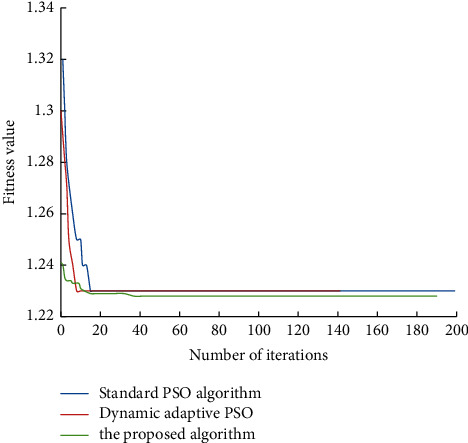
Comparison diagram of changes in fitness function values.

**Figure 6:**
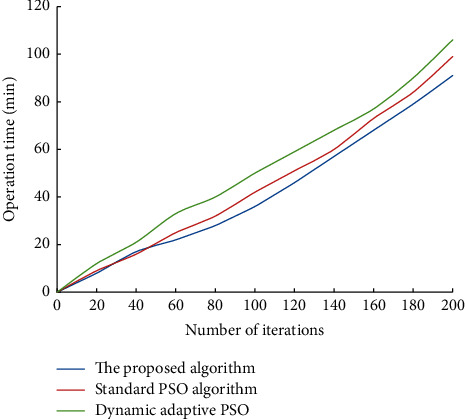
Comparison of operation time of different algorithms.

**Figure 7:**
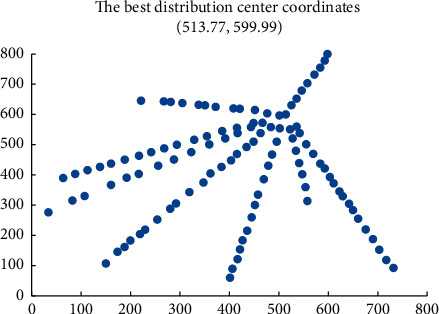
Site selection results of community amateur sports training facilities.

The proposed algorithm is compared with the standard PSO algorithm (*c1* and *c2* are constant, and inertia weight decreases linearly). The dynamic adaptive PSO algorithm runs the corresponding program. The change of fitness function value is shown in Figure 5. A comparison of the operation time of different algorithms is shown in Figure 6. The location results of community amateur sports training facilities are shown in Figure 7.

The improved PSO algorithm based on the dual mechanism proposed in the paper is used to solve the location problem of community amateur sports training facilities, which has good convergence. The number of iterations to obtain the optimal solution is about 40 times, the best distribution center coordinates (513.77, 599.99), and the minimum total cost of construction and later distribution is 1228 million yuan. The standard PSO algorithm and the dynamic adaptive PSO algorithm can not converge to the global optimum, although the solution results are close to the solution results of the former. This paper constructs the mathematical model of community amateur sports training facility location and solves the optimal community amateur sports training facility location scheme through the improved PSO algorithm. The obtained scheme can significantly reduce the total cost to the minimum. Therefore, the PSO algorithm based on a dual mechanism has achieved the desired results in solving the location problem of community amateur sports training facilities, which has a very positive significance.

## 4. Conclusion

The traditional PSO is dynamically self-adjusted and optimized from inertial weight and acceleration factors, and dynamically changing control factors are added to improve the efficiency. Results show that the performance of PSO is improved significantly by the dynamic adaptive adjustment of multiple parameters, and the overall optimization ability of the improved is enhanced. The improved PSO can be used to solve nonlinear and complex problems such as path planning and automatic control. Through the application of the sports training facility location model, which proved the accuracy of the location selection model and adaptive weighting based on improved multiobjective PSO to solve complex optimization problems the effectiveness of the adaptive weighting based on improved multiobjective PSO to improve the easy to fall into part optimum, the disadvantage of poor robustness, the model is applied to sports training facility location problem solving, it has good dynamic observation and convergence. In the next step, we will continue to optimize the algorithm and apply the model to solve other problems, such as commercial location and constantly optimize and improve it.

## Figures and Tables

**Table 1:** Satisfaction survey.

Distance (m)	Time spent (min)	Satisfaction
[0, 500]	[0, 8]	Very satisfied
[500, 1000]	[8, 15]	Satisfied
[1000, 1500]	[15, 23]	Basically satisfied
[1500, 2000]	[23, 30]	Dissatisfied

**Table 2:** Community location coordinates and fitness demand.

Community serial number	Coordinate	Demand (person-time)
1	(200, 1500)	125
2	(600, 800)	132
3	(1500, 300)	128
4	(2000, 1500)	112
5	(600, 2000)	116
6	(1000, 2500)	119
7	(3000, 1500)	111
8	(1500, 200)	135

**Table 3:** Distribution of construction costs.

Sports facilities serial number	Position coordinates	Construction cost (yuan)
1	*x* ∈ [200, 3000), *y* ∈ (0, 1500]	30000
2	*x* ∈ (1500, 3000), *y* ∈ (0, 3000]	35000
3	*x* ∈ [200, 1500], *y* ∈ (1500, 3000]	40000
4	*x* ∈ (1500, 3000], *y* ∈ [200, 1500]	25000

**Table 4:** Total operating cost analysis table.

Coordinate	Construction cost (yuan)	Demand (person-time)	Wreck a cost (yuan)	Total operating cost (yuan)
(200, 1500)	30000	125	250	30250
(600, 800)	30000	132	264	30264
(1500, 300)	35000	128	256	35256
(2000, 1500)	35000	112	224	35224
(600, 2000)	40000	116	232	35232
(1000, 2500)	40000	119	238	35238
(3000, 1500)	25000	111	222	35222
(1500, 200)	25000	135	270	35270

## Data Availability

The labeled dataset used to support the findings of this study are available from the corresponding author upon request.
